# HMGN1 and R848 Synergistically Activate Dendritic Cells Using Multiple Signaling Pathways

**DOI:** 10.3389/fimmu.2018.02982

**Published:** 2018-12-18

**Authors:** Md Masud Alam, De Yang, Anna Trivett, Thomas J. Meyer, Joost J. Oppenheim

**Affiliations:** ^1^Cancer and Inflammation Program, Frederick National Laboratory for Cancer Research, Center for Cancer Research, National Cancer Institute, Frederick, MD, United States; ^2^Advanced Biomedical Computational Science, Frederick National Laboratory for Cancer Research sponsored by the National Cancer Institute, National Institutes of Health, Bethesda, MD, United States

**Keywords:** TLR, HMGN1, R848, IFN, NF-κB, MAPK, Th1 polarization, dendritic cell

## Abstract

High mobility group nucleosome-binding protein 1 (HMGN1 or N1) is a Th1-polarizing alarmin, but alone is insufficient to induce antitumor immunity. We previously showed that combination of N1 and R848, a synthetic TLR7/8 agonist, synergistically activates dendritic cells (DCs) and induces therapeutic antitumor immunity, however, it remained unclear how N1 and R848 synergistically activate DCs. Here, we show that co-stimulation with N1 and R848 of human monocyte-derived DCs (MoDCs) markedly upregulated DC's surface expression of CD80, CD83, CD86, and HLA-DR, as well as synergistic production of pro-inflammatory cytokines including IL-12p70, IL-1β, and TNF-α. This combination also synergistically activated NF-κB and multiple MAPKs that are involved in DC maturation. Moreover, N1 and R848 synergistically increased nuclear translocation of interferon (IFN) regulatory transcription factors (e.g., IRF3 and IRF7) and promoted the expression of type 1 IFNs such as IFN-α2, IFN-α4, and IFN-β1. Similar signaling pathways were also induced in mouse bone marrow-derived DCs (BMDCs). RNA-seq analysis in human MoDCs revealed that N1 plus R848 synergistically upregulated the expression of genes predominantly involved in DC maturation pathway, particularly genes critical for the polarization of Th1 immune responses (e.g., *IL12A, IL12B*, and *IFNB1*, etc.). Overall, our findings show that (1) N1 synergizes with R848 in activating human and mouse DCs and (2) the synergistic effect based on various intracellular signaling events culminated in the activation of multiple transcriptional factors. These findings have important implications for future clinical trials since N1 and R848 synergistically promoted optimal Th1 lineage immune responses resulting in tumor rejection in mice.

## Introduction

Immunotherapy is based on the hypothesis that patients' immune systems can be stimulated to attack the malignant tumor. This idea is based on Coley's observations in 1891 that injection of bacteria or bacterial products into cancer patients resulted in an excellent antitumor effect on bone and soft tissues sarcomas (1). Although the mechanisms by which bacteria or bacterial products induced suppression of malignant tumors were not identified in Coley's time, these products are presumably sensed by Toll-like receptor (TLR) signaling pathways of host cells, as discovered by more recent scientific advances. These TLR-mediated signaling pathways play an important role in the induction of innate and adaptive immune responses. Thirteen distinct TLRs have been discovered in mammals and dendritic cells (DCs) expressing these receptors are activated by TLR agonists (2, 3). DCs in turn activate T cells by delivering antigenic signals, co-stimulatory signals as well as cytokine signals. We have therefore chosen to use TLR ligands to treat tumors in mice based on their great capacity to stimulate DCs.

HMGN1 (High mobility group nucleosome-binding protein 1, N1), an endogenous protein that is an alarmin activator of TLR4, has been shown to be a potential inducer of antitumor immunity (4) and a consistent potent immune stimulating alarmin in the induction of adaptive immune responses (4–6). N1 activates DCs in a MyD88 (myeloid differentiation primary response gene 88) and TRIF (TIR domain containing adaptor protein inducing IFN-β) dependent manner (4). It is a unique alarmin that binds to a site distinct from where LPS interacts on MD2 in the TLR4 receptor complex. Furthermore, N1 still has a minor activating effects on cells from C3H/HeJ and TLR4 knockout mice using an unidentified pathway. In addition, N1 induces migration of DCs *in vitro* and recruitment *in vivo* through GiPCR mediated signaling pathways (our unpublished data). N1 enhances antigen-specific immune response with increased production of IL-12 and induces T-helper type 1 (Th1) polarization in mouse models (4, 7). IL-12 is a DC-derived cytokine inducer of IFN-γ production and a potent inducer of Th1 responses, that bridges innate and adaptive immune responses (8). N1 contributes to the induction of antitumor immune responses against mouse thymoma as well as melanoma and can be used as an effective prophylactic anticancer immunoadjuvant (9). However, N1 alone was unable to therapeutically cure established mouse tumors. Since combined activation of multiple TLR pathways can potentially result in more potent agonistic effects on host immune responses (10), we therefore screened various other TLR agonists to determine the one that could synergize with N1 for the activation of DCs, and established that R848, when combined with N1, resulted in maximal activation of DC as well as induction of antitumor immunity (11).

R848, an antiviral imidazoquinoline derivative, activates immune cells through TLR7 and/or TLR8 in a MyD88-dependent manner (12). Murine TLR7 is clearly sensitive to R848 because TLR7^−/−^ mice fail to respond to R848, even though TLR8 is present in the immune cells (13, 14). TLR8^−/−^ murine DCs have increased TLR7 expression and ultimately manifest a potent response to R848 (13, 14). No specific ligand for murine TLR8 is known. Nevertheless, a study of TLR8^−/−^ mice indicated that TLR8 regulates TLR7 expression and protects mice from autoimmunity, since TLR8^−/−^ mice, but not TLR8^−/−^ TLR7^−/−^ mice, developed features of lupus-like syndrome (15). In mouse studies, R848 was shown to act as an adjuvant able to promote an adaptive immune response to co-administered antigens (16).

The combination of N1 plus R848 can synergistically induce the maturation of human and mouse DCs (11), however, it remains unknown how N1 plus R848 activates DCs in a synergistical manner. Here we show that the synergistic properties of N1 plus R848 on DC maturation are elicited through synergistic activation of nuclear factor (NF)-κB, mitogen activated protein kinases (MAPK), interferon regulatory factor (IRF)-3 as well as IRF7 signaling pathways. DCs exhibit synergistic upregulation of co-stimulatory molecules (CD80, CD83, and CD86) and produced high levels of pro-inflammatory cytokines (IL-12p70, IL-1β, and TNF-α) as well as type 1 IFN (such as IFN-α2, IFN-α4, and IFN-β1) in response to treatment with N1 plus R848. RNA-seq and allogeneic mixed lymphocyte reaction (MLR) studies revealed that treatment with N1 plus R848 synergistically activated human DCs toward the direction of preferentially inducing Th1 polarization. Our study shows for the first time that synergistic activation of NF-κB, MAPK, IRF3, and IRF7 transcription factors are responsible for the synergistic pro-inflammatory cytokine as well as type 1 IFN production in response to combined stimulation with N1 plus R848 in both human monocyte derived (Mo)-DCs as well as mouse bone marrow derived (BM)-DCs.

## Materials and Methods

### Mice and Reagents

Male wild-type C57BL/6 mice, 7–8 weeks old were provided by the animal production facility of the National Cancer Institute (NCI). National Cancer Institute (NCI) is approved by the American association for the accreditation of laboratory animal care and follows the public health service policy for the care and use of laboratory animals. Animal care was provided in accordance with the procedures outlined in the guide for care and use of laboratory animals (National research council, Washington DC). Recombinant human or mouse GM-CSF, IL-4 were purchased from PeproTech. R848 was obtained from Invivogen. Recombinant N1 was generated in insect cells and purified as previously described (4, 17).

### Isolation and Purification of Cells

Human peripheral blood cell samples were obtained from healthy donors (Transfusion medicine department, Clinical center, National institutes of health, Bethesda, MD, with an approved human subject agreement) by leukopheresis. Peripheral blood mononuclear cells (PBMC) were isolated by Ficol-Hypaque (Sigma-Aldrich, St-Louis, MO) density gradient centrifugation. Monocytes and CD4+ T cells were purified (>95%) from PBMC with the use of MACS CD14 monocyte and CD4 T isolation kits (Miltenyl Biotech, Auburn, CA) according to the manufacturer instructions (4, 5). Mouse bone marrow derived hematopoietic progenitor cells (HPCs) were prepared from C57BL/6 wild type mice (Male 7–8 weeks old) by flushing from femur and tibia with the depletion of red blood cell (RBCs) by ammonium chloride treatment [ACK lysing buffer, Quality Biological] (18).

### Generation and Stimulation of Human MoDCs and Mouse BMDCs

Human MoDC and mouse bone marrow-derived DC (BMDC) generation were performed as described previously (4). Briefly, human MoDCs were generated by culturing purified monocytes at 5 × 10^5^ cells/ml in complete RPMI 1,640 medium (RPMI 1,640 medium [Mediatech Inc] supplemented with 10% FBS [GemCell], 2 mM glutamine [Lonza], 25 mM HEPES [Quality Biological], 100 U/ml penicillin [Lonza], 100 μg/ml streptomycin [Lonza], and 50 μM of 2-Mercaptoethanol [Sigma]) containing 50 ng/ml human GM-CSF and IL-4 at 37°C in a humidified CO_2_ (5%) incubator for 5 days with 50% of the culture medium replaced with prewarmed fresh complete RPMI 1,640 medium containing human GM-CSF and IL-4 at the same concentration on days 3. On the 5th day of culture, cells in suspension were harvested and used as immature MoDCs (purity almost 99.99% observed under microscopy) for subsequent experiments. Mouse BMDCs were generated by culturing mouse HPCs isolated from the femurs and tibias in complete RPMI 1,640 containing 20 ng/ml mouse GM-CSF for 6 days. On the 2nd and 4th days of culture, non-adherent cells were removed by gentle pipetting and the remaining adherent cells were cultured in the complete medium containing mouse GM-CSF at the same concentration as indicated above. On the 6th day of culture, cells in suspension were harvested as immature DCs and used for subsequent experiments. Immature human MoDCs and mouse BMDCs were stimulated with N1 and/or R848, for 30 min (') to 48 h at 37°C in a CO_2_ incubator before being analyzed for phenotype, antigen-presenting function, and signaling.

### Assessment of Human MoDC and Mouse BMDC Phenotypes Through Surface FACS Staining

Stimulated and non-stimulated DCs (10^6^ cells/samples) were first washed three times with fluorescence activated cell sorter (FACS) buffer (PBS [Mediatech Inc], 2% FBS, 0.05% NaN_3_ [ThermoFisher Scientific], pH 7.4) and blocked for 10 min at 4°C in FACS buffer containing 2% human AB serum (Sigma Aldrich) and/or 2% normal mouse serum (C57BL/6 mouse) (11). Subsequently, the cells were stained with monoclonal antibodies against human CD80 (BD Pharmingen, FITC conjugated IgG1, κ, clone L307.4), CD83 (BD Pharmingen, PE conjugated IgG1, κ, clone HB15e), CD86 (BD Pharmingen, PerCP-Cy5.5 conjugated IgG1, κ, clone 2331), HLA-DR (Biolegend, PB conjugated IgG2b, κ, clone LN3) or mouse CD80 (BD Pharmingen, eFluor 450 conjugated IgG, κ, clone 16-10A1), CD86 (BD Pharmingen, FITC conjugated IgG2a, κ, clone GL1), I-A/E (Biolegend, PE/Cy7 conjugated IgG2b, κ, clone M5/114.15.2) with the isotype matched control antibodies at 4°C for 20–30 min. Then the cells were washed once with flow cytometry buffer and twice with PBS, suspended in PBS and analyzed the expression of surface molecules on LSR II flowcytometry (BD FACSCalibur). Data were analyzed by FlowJo software version 10.1.

### Measurement of Cytokine Levels in Culture Supernatants of Human MoDCs and Mouse BMDCs

Multiple cytokines in the culture supernatants of human and mouse DCs were measured using V-PLEX (IL-1β, TNF-α, and IL-12p70) and U-PLEX (IFN-γ) ultrasensitive plate assays (Meso Scale Discovery) (19). The plates were analyzed using a sector image 2,400 (Meso Scale Discovery) according to the manufactures protocol. The detection limits of the human cytokines are IL-12p70: 0.117pg/ml; IL-1β: 0.137 pg/ml; and TNF-α: 0.0859 pg/ml and the limits for the mouse cytokines are IL-12-p70: 4.90 pg/ml; IL-1β: 0.0793 pg/ml; and TNF-α: 0.0327 pg/ml. The data comparing the means of the stimulated samples to those of non-stimulated control were analyzed by GraphPad Prism version 7.

### MLR

Allogenic MLR was performed as previously described (5). Briefly, human MoDCs (4 × 10^6^ cells/8 ml/well) were cultured in 6-well plates and treated with N1 and/or R848 for 2 days at 37°C in a humidified air with a 5% CO_2_. The treated DCs were co-cultured with purified allogenic CD4+ T (10^5^ cells/well) cells at 1:250, 1:750, 1:2,250, 1:6,750, and 1:20,250 ratios in a complete medium (0.2 ml/well) in a triplicate in 96-well plate for 4 days at 37°C. After 4 days of co-culture, the cells were pulsed with 1.0 μCi/well [^3^H]-TdR (New England Nuclear) for an additional 18 h before harvesting. ^3^H-TdR incorporation was measured with microbeta counter (Wallace, Gaithersburg, MD) for the proliferative response of allogenic CD4+ T cells. Alternatively, the supernatants of the MLR culture in a complete medium (0.4 ml/well) in 48-well plates at 3 days were collected for cytokine quantitation. Data were analyzed by GraphPad Prism version 7.

### Analysis of T-bet and Gata-3 Transcription Factor by Intracellular FACS Staining

Intracellular staining was performed according to the referred eBiosciences staining intracellular protocol assays for FACS. Briefly, human MoDCs (4 × 10^6^ cells/8 ml/well) were cultured in 6-well plates and treated with N1 and/or R848 for 2 days at 37°C. The treated DCs were co-cultured with allogenic CD4+ T (10^6^ cells/well) cells at 1:250 ratio in a complete medium (0.4 ml/well) in triplicate in 48-well plates for 3 days at 37°C in a humidified air containing 5% CO_2_, with the addition of GolgiPlug cell stimulation cocktail (composed of Phorbol ester, Ionomycin, PMA, Brefeldin A and Monensin from Tonbo Biosciences) for the last 6 h of co-culture (9). Cells were collected, washed with FACS buffer, blocked with 2% human AB serum for 10 min and stained with monoclonal antibodies against human CD4 (Tonbo biosciences, APC-cy7 conjugated IgG1, κ, clone RPA-T4) for 30 min at room temperature. Cells were washed (with FACS buffer once and PBS twice), fixed with intracellular fix/perm buffer (Tonbo bioscience; cat. No. TNB-1020-L050) for 18 h in dark at 4°C, then washed by adding permeabilization buffer (Tonbo bioscience; cat. No. TNB-1213-L150) to the fixed cells, centrifuged and blocked by 2% normal mouse serum (made in permeabilization buffer) for 10 min. Subsequently, Cells were incubated with monoclonal antibodies against human T-bet (Biolegend, PB conjugated IgG1, κ, clone 4B10) and Gata-3 (BD Pharmingen, PE conjugated IgG1, κ, clone L50-823) in permeabilization buffer for 1 h at room temperature. After three washes with permeabilization buffer, the samples were resuspended in 1% paraformaldehyde in PBS and data were acquired on a LSRII flow cytometer. Flow cytometry data were analyzed using FlowJo software version 10.1.

### SDS-PAGE and Western Blot Analysis

Sodium dodecyl sulfate-polyacrylamide gel electrophoresis (SDS-PAGE) and western blot analysis were performed as described previously (20). Briefly, serum starved human MoDCs and mouse BMDCs were incubated for 6 h with individual or combined ligands for a period as specified. The treated and untreated cells were lysed in SDS whole cell lysis buffer composed of 62.5 mM Tris-HCl, pH 6.8 (Quality biological) at 25°C, 2% wt/vol SDS (Quality biological), and 40 mM dithiothreitol (Sigma aldrich) at 10^7^ cells/ml for 30 min at room temperature. Nuclear and cytoplasmic proteins were collected using NE-PER extraction reagents according to manufacturer's instructions (ThermoFisher Scientific) and quantitated by Pierce BCA protein assay kits (ThermoFisher Scientific). NuPAGE LDS sample buffer (ThermoFisher Scientific) were added to these protein lysates. 1 × NuPAGE MOPS SDS running buffer was used as an electrode buffer. After transfer of separated proteins onto polyvinylidene difluoride (PVDF) membranes (Immobilon, Millipore), the membrane were rinsed with tris-buffered saline (Quality biological) containing 0.05% tween 20 (Sigma aldrich) (TBST), blocked with blocking buffer (1 × TBST containing 5% non-fat milk [BioRad]) at room temperature for 1 h and incubated overnight at 4°C with antibodies (1:1,000 diluted in blocking buffer) against phospho-p38 (Cell signaling technology [CST], T180/Y182), phospho-JNK (CST, T183/185), phospho-CREB (CST, 87G3), phospho-c-Jun (CST, 60A8), phospho-I-κBα (CST, 14D4), phospho-p65 (CST, S536), IRF3 (CST, D83B9), IRF7 (CST, D2A1J, human specific), and IRF7 (Abcam, ab109255, mouse specific) as the first antibody. After washing with TBST, the membranes were reacted with horseradish peroxidase (HRP)-conjugated goat anti-rabbit IgG (CST, 7074S) as the secondary antibody (1:2,000 diluted in blocking buffer), washed and developed in the SuperSignal West Dura Extended Duration Substrate (ThermoFisher Scientific). The images were collected using the G BOX Chemi systems (Syngene). Subsequently, the same membrane was stripped by blot stripping buffer (ThermoFisher Scientific) and probed consecutively in the same manner using p38 (CST, 9212S), JNK (CST, 9252S), CREB (CST, 48H2), c-Jun (CST, 60A8), I-κBα (CST, 44D4), lamin B1 (Abcam, D4Q4Z), and GAPDH (CST, 14C10) as the first antibodies. The immunoreactive bands were quantified by densitometry with ImageJ 1.52a software (National Institutes of Health, USA) according to the manufacturer's instructions.

### RNA Extraction, cDNA Synthesis and Real-Time (RT)-PCR

Total RNA from the treated and control DCs was isolated using TRIzol reagent (Invitrogen Life Technologies) according to manufacturer's instructions. The RNA was purified using RNeasy Micro Kit (Qiagen) with an on-column DNase digestion to eliminate possible genomic DNA contamination. Purified RNA (500 ng) was reverse-transcribed to cDNA with QuantiTect Reverse Transcription kit (Qiagen). Twenty nanograms of cDNA was used for qRT-PCRs. qRT-PCRs were performed with a RT^2^ DNA SYBR green ROX kit (Qiagen) on ABI 7,500 Fast RT-PCR system to measure the expression of human and mouse cytokines using specific primers obtained from Qiagen (Table S1 in Supplementary Material). ΔCt values were obtained by deducting the raw cycle threshold (Ct values) obtained from β-Actin mRNA, the internal standard, from the Ct values obtained for investigated genes. For graphical representation, data are expressed as fold mRNA level increase compared to the expression level in unstimulated cells using GraphPad Prism version 7 (11).

### RNA-seq Analysis

Total RNA of stimulated human MoDCs from three donors was isolated using the QIAGEN RNeasy kit according to manufacturer's instructions. The integrity was checked by the RNA integrity number (RIN) value and samples that passed the quality control check were used for sequencing at the NCI, CCR sequencing facility. Briefly, 400 ng of total RNA was used as the input to an mRNA capture with oligo-dT coated magnetic beads. The mRNA is fragmented, and then a random-primed cDNA synthesis is performed (Illumina, Inc.). The resulting double-strand cDNA is used as the input to a standard Illumina library prep with end-repair, adapter ligation and PCR amplification being performed yielding a sequencing ready library. The final purified product is then quantitated by qPCR (Kapa Biosystems) before cluster generation. Samples were pooled and sequenced on one HiSeq 4,000 run using Illumina TruSeq Stranded Total RNA Kit RS-122-2201. The HiSeq RT Analysis software (RTA v1.18.64) was used for base calling. The Illumina bcl2fastq v2.17 software was used to demultiplex and convert binary base calls and qualities to FASTQ format. RNA-seq NGS-datasets were processed using the CCBR Pipeliner utility (https://github.com/CCBR/Pipeliner). Briefly, reads were trimmed of low quality bases and adapter sequences were removed using Trimmomatic v0.33 (21). Mapping of reads to the GRCh37 (hg19) human reference genome was performed using STAR v2.5.2b in 2-pass mode (22). Then, RSEM v1.3.0 was used to quantify gene-level expression, with counts normalized to library size as counts-per-million (23). Finally, limma-voom v3.34.5 was used for quantile normalization and differential expression (24). The data discussed in this publication have been deposited in NCBI's Gene Expression Omnibus (25) and are accessible through GEO Series accession number GSE121607 (https://www.ncbi.nlm.nih.gov/geo/query/acc.cgi?acc=GSE121607). Genes were considered variable if significantly differentially expressed relative to sham (adjusted *p*<*0.05*) and absolute fold change relative to sham was ≥2.0. Pathway analysis was performed using Ingenuity Pathway Analysis (26) and a pathway was considered significantly activated/deactivated if the absolute activation z-score in all contrasts was ≥2.0. Gene ontology (GO) term analysis was performed using ClueGO (27). Expression data were visualized as heat maps using ClustVis (28).

### Statistical Analysis

All experiments were performed at least three times with similar results. The statistical significance between groups were analyzed by using Microsoft Excel and GraphPad Prism version 7. Data are expressed as the average (mean ± SD) of multiple experiments. Statistical significance of differences among the groups was determined by an ordinary one-way ANOVA (non-parametric) followed by Tukey's *post hoc* test with a threshold set as *p*<*0.05*.

## Results

### N1 and R848 Dose Dependently Activate Human MoDCs

**Figure 1:**
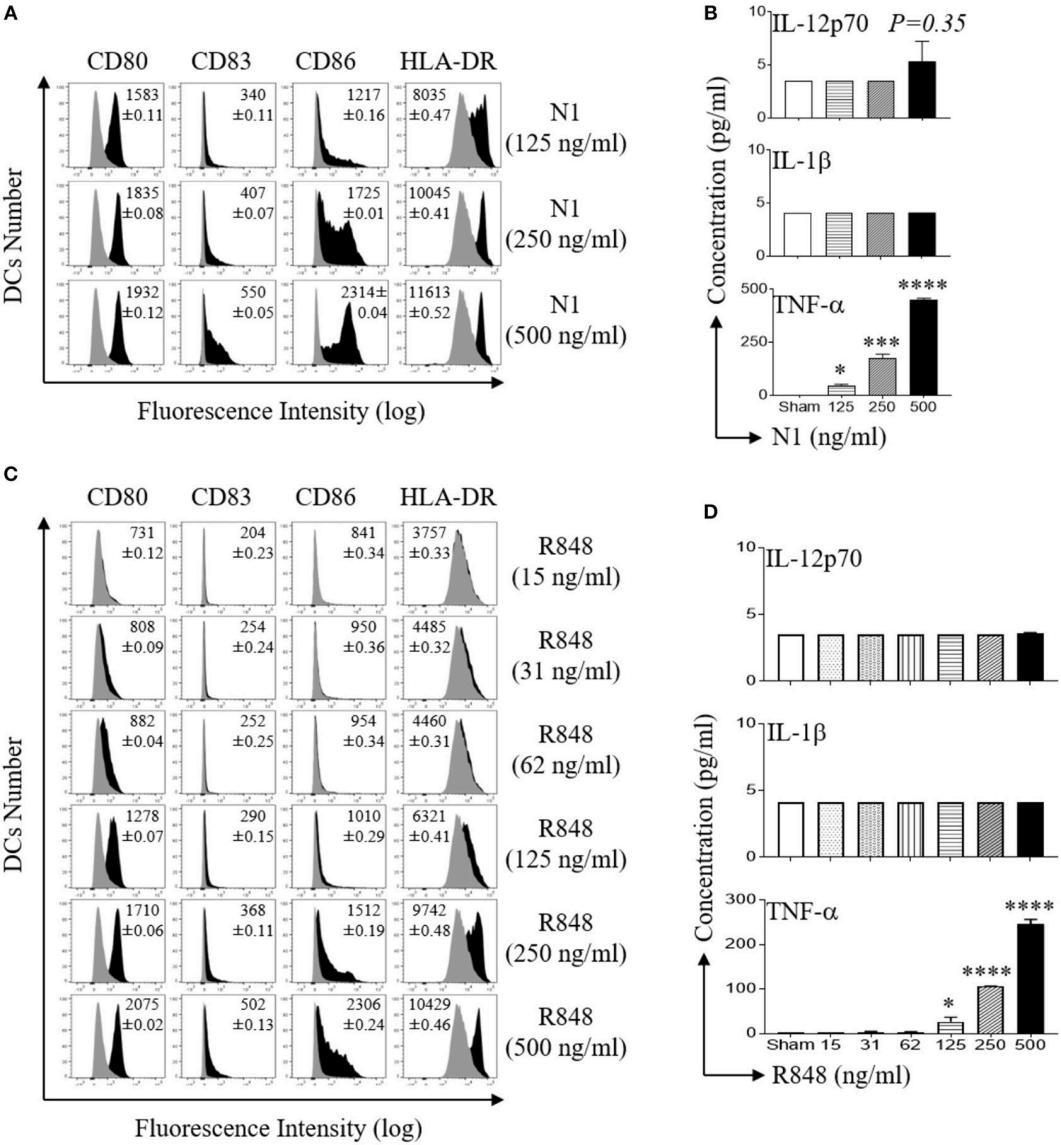
N1 and R848 dose dependently induce phenotypic maturation of human MoDCs. **(A,C)** Human MoDCs were incubated at 5 × 10^5^ cells/ml in the absence (sham) or presence of indicated concentrations of recombinant N1 or R848 for 48 h before they were immunostained and analyzed by flow cytometry for the expression of the indicated surface molecules. Shown are the overlay histograms (gray area = sham-treated) of one experiment, with the average relative fluorescence intensity (geometric mean ± SD) of three independent experiments inscribed. **(B,D)** Cytokine levels in the culture supernatants of DCs treated with N1 or R848 at indicated concentrations for 48 h were quantitated by cytokine array (mean ± SD; *n* = 3). *p<*0.05*, ***p<*0.001* and ****p<*0.0001* when compared with the sham treatment according to one-way ANOVA followed by Tukey's *post hoc* test.

Activation of DCs results in the acquisition of a mature phenotype with upregulation of surface expression of co-stimulatory molecules and the production of pro-inflammatory cytokines (29). We have previously reported that recombinant protein, N1 and a synthetic imidazoquinoline, R848 synergistically activates DCs (4, 11). To determine the optimal doses of N1 and R848 to activate human MoDCs, we stimulated pure primary human immature DCs generated from CD14+ monocytes for 48 h, previously determined to be an optimal duration of incubation. We determined the minimal to maximal doses for each of the stimulants (4). The results showed that N1 (125, 250, and 500 ng/ml) and R848 (15–500 ng/ml) dose-dependently upregulated co-stimulatory (CD80, CD83, and CD86) as well as MHC class II (HLA-DR) molecules, indicating that these human MoDCs acquired phenotypic markers of maturation (Figures 1A,C). N1- and R848-treated human MoDCs each produced more TNF-α protein in a dose-dependent manner but did not elevate IL-12p70 and IL-1β levels (Figures 1B,D). The production of increased TNF-α and induction of phenotypic markers identified the doses yielding optimal responses by activated human MoDCs. Stimulation with 1 μg/ml of reagents as previously reported resulted in suboptimal activation of human MoDCs (4). Therefore, N1 and R848 each induced dose-dependent activation of human MoDCs with 500 ng/ml yielding maximal effect.

### N1 and R848 Result in Dose Dependent Synergistic Activation of Human MoDCs

**Figure 2:**
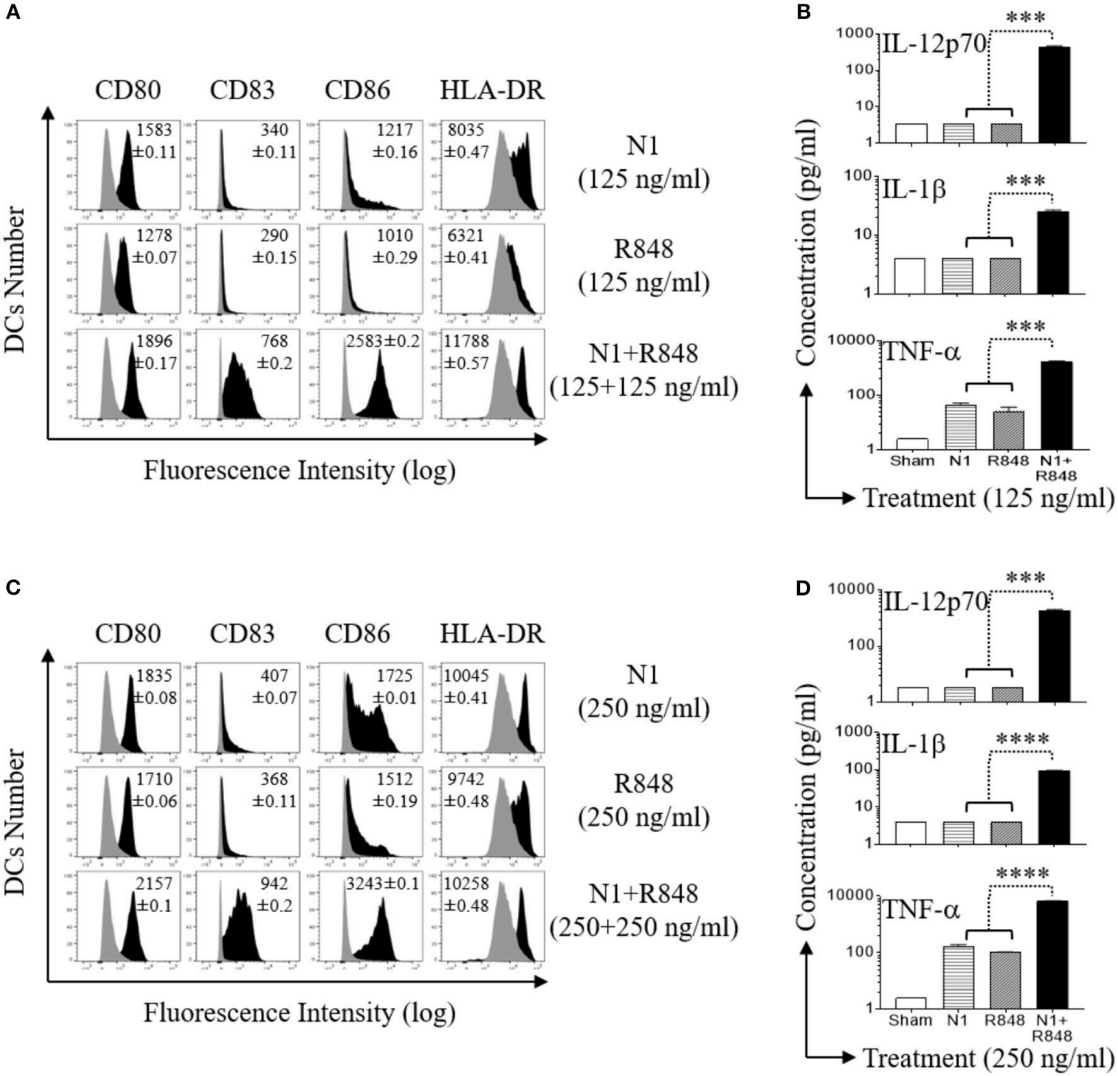
N1 and R848 synergistically induce phenotypic maturation of human MoDCs. Human MoDCs were incubated at 5 × 10^5^ cells/ml in the absence (sham) or presence of various concentrations of N1 and/or R848 for 48 h before they were analyzed for surface marker expression and cytokine production. **(A,C)** The expression of indicated surface molecules is shown as overlay histograms (gray area = sham-treated) of one donor, with the average relative fluorescence intensity (geometric mean ± SD) of three independent donors inscribed. **(B,D)** Cytokine levels in the culture supernatants were quantitated by cytokine array (mean ± SD; *n* = 3). ***p<*0.001*, and ****p<*0.0001* according to one-way ANOVA followed by Tukey's *post hoc* test.

Since N1 together with R848 synergize in upregulating the surface expression of co-stimulatory molecules on DCs and synergistically promote the production of pro-inflammatory cytokines by DCs (11), it is essential to determine the doses resulting in optimal synergistic activation of DCs. We therefore determined the effects of different dose ranges of N1 (125, 250, and 500 ng/ml) and R848 (15–500 ng/ml) in combination on human MoDCs response at 48 h (Figures S2A,B, S3A,B, and S4A,B in Supplementary Material, respectively). Concentrations of 250 ng/ml N1 with 250 ng/ml R848 yielded maximal synergistic upregulation of cell surface expression of co-stimulatory (CD80, CD83, CD86, and HLA-DR) molecules (Figures 2A,C) as well as production of pro-inflammatory cytokine (IL-12p70, IL-1β, and TNF-α) proteins (Figures 2B,D) and mRNA levels (Figures S1A–C in Supplementary Material, respectively). The doses of N1 plus R848 at 250 ng/ml induced about 4-fold higher production of pro-inflammatory cytokines by activated human MoDCs than stimulation by a concentration of 125 ng/ml. Analogous, but suboptimal synergistic responses were also obtained using other doses of N1 together with R848 (Figures S2A,B, S3A,B, S4A,B, respectively). Although N1 and R848 signaling alone induced only increases in TNF-α levels, the combination of 250 ng/ml each of N1 plus R848 led to robust production of IL-12p70, IL-1β, and TNF-α by human MoDCs. Therefore, 250 ng/ml N1 with 250 ng/ml R848 maximally activated human MoDCs. We subsequently used these doses to obtain signal transduction responses.

### N1 and R848 Induced Synergistic Th1 Polarization of Allogeneic Naïve Human CD4+ T Cells

**Figure 3:**
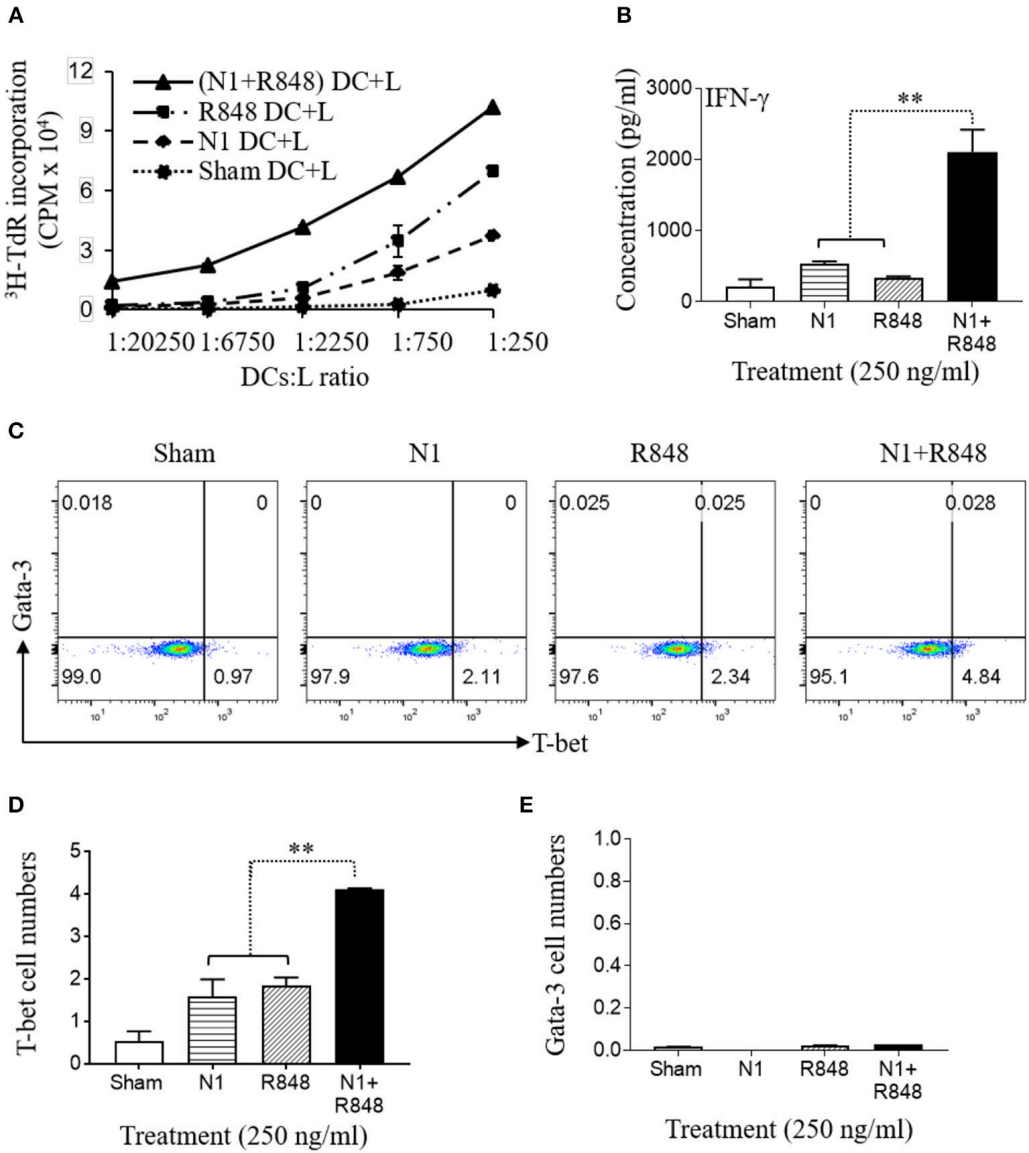
N1 and R848 synergistically induce functional maturation of human MoDCs. **(A–E)** Human MoDCs cultured in the presence or absence of N1 (250 ng/ml) and/or R848 (250 ng/ml) for 48 h were incubated in triplicate with allogenic human peripheral blood CD4+ T lymphocytes (L) (10^5^ cells/well). **(A)** On day 4, 1 μCi of [^3^H]TdR was added to each well to pulse the cultures for another 18 h. The proliferation was measured as the average (mean ± SD) of [^3^H]TdR incorporation (CPM) of triplicate wells. **(B)** IFN-γ production by CD4+ T cells co-cultured with DCs (DCs:L = 1:250) for 3 days (mean ± SD, *n* = 3). **(C–E)** The expression of T-bet and Gata-3 by CD4+ T cells co-cultured with DCs (DCs:L = 1:250) for 3 days were measured by flow cytometry. **(C)** Representative dot plots. **(D,E)** The average (mean ± SD) of triplicate wells. Shown is the results of one representative of three independent experiments obtained from different donors. **p<*0.01* according to one-way ANOVA followed by Tukey's *post hoc* test.

Previously, it has been reported that N1- or R848-treated DCs have the capacity to stimulate the proliferation of allogeneic naïve CD4+ T cells (4, 30, 31). To analyze the effect on functional maturation of N1- plus R848-treated human MoDCs, we co-cultured the human MoDCs stimulated with N1 at 250 ng/ml together with R848 at 250 ng/ml plus allogeneic naïve CD4+ T lymphocytes (L) at different human MoDCs/L ratio for 5 days to determine the proliferative response of CD4+ T (L) cells in MLR's. Human MoDCs treated with the combination of N1 plus R848 synergistically stimulated [^3^H]TdR incorporation by allogenic CD4+ T cells compared with N1 or R848 alone. Therefore, human MoDCs treated with these TLR ligands exhibited synergistically enhanced capacity to antigenically activate CD4+ T cells to induce an MLR (Figure 3A).

IL-12p70 heterodimer, composed of p35 and p40 chains, promotes the development of naïve T cells into Th1 cells and resultant IFN-γ production (32). Therefore, the cells were cultured for 3 days at a human MoDCs/L ratio of 1:250 to determine the levels of IFN-γ as well as Th1 polarizing transcription factor T-bet. Treatment of DCs with N1 plus R848 resulted in a remarkable synergistic upregulation of IFN-γ cytokine production (Figure 3B) and the expression of the T-bet transcription factor by the CD4+ T cells, while each stimulant alone was only minimally active in this respect (Figures 3C–E). The above results indicated that the high levels of cytokines and particularly IL-12p70 secretion by DCs activated with N1 plus R848 synergistically polarize CD4+ T cells toward a Th1 response to a much greater extent than activation with single TLR agonists.

### Stimulation of Human MoDCs by N1 Plus R848 Synergistically Activates NF-κB and MAPK Pathways

**Figure 4:**
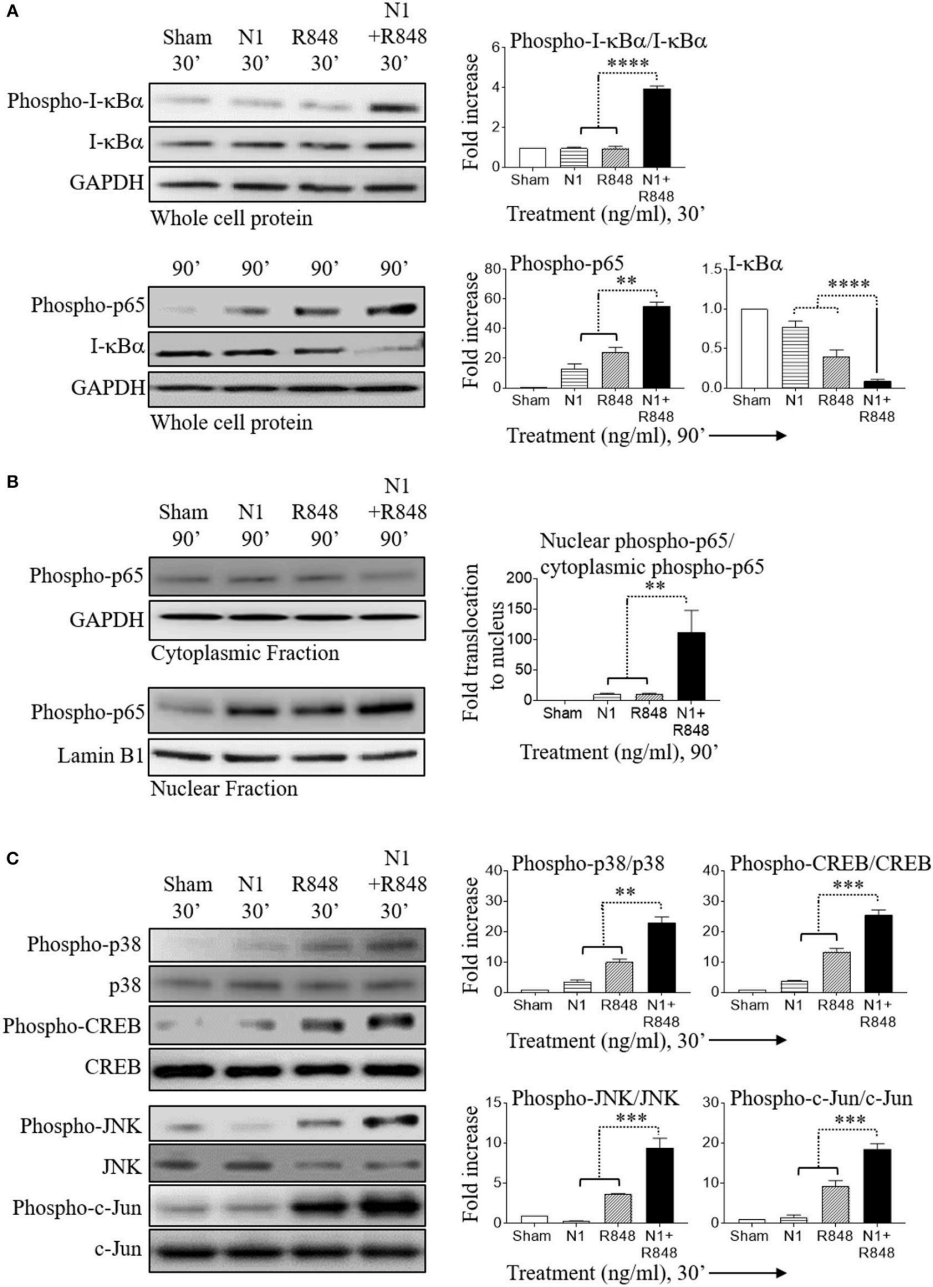
N1 and R848 synergistically activate NF-κB and MAPK signaling in human MoDCs. Serum-starved human MoDCs (2 × 10^6^ cells/ml) were treated for 0–90 min with N1 (250 ng/ml) and R848 (250 ng/ml) before they were solubilized in lysis buffer (10^6^ MoDCs/0.1 ml) to obtain whole cell lysates or in buffer (2 × 10^6^ MoDCs/0.1 ml) for analysis of the translocation of proteins from the cytoplasmic to nuclear cell fractions. An identical amount of protein was loaded and separated by electrophoresis to detect the levels of phospho-I-κBα, I-κBα, phospho-p65, phospho-p38, p38, phospho-CREB, CREB, phospho-JNK, JNK, phospho-c-Jun, c-Jun, and GAPDH in whole cell lysates **(A,C)** or **(B)** cytoplasmic fraction of phospho-p65, GAPDH and nuclear fraction of phospho-p65, lamin B1. Quantitation of band intensities from three donors using ImageJ software were normalized against GAPDH or lamin B1. **p<*0.01*, ***p<*0.001*, and ****p<*0.0001* according to one-way ANOVA followed by Tukey's *post hoc* test.

In resting cells, the transcription factor NF-κB exists mainly in the cytosol where it binds to I-κBα. Upon cell activation, I-κBα is phosphorylated, ubiquitinated, and then degraded leading to the release of NF-κB, which subsequently translocate to the nucleus and causes inflammatory gene transcription (33, 34). Therefore, activation of the NF-κB pathway was determined using degradation of I-κBα as a readout. Stimulation with N1 at 250 ng/ml plus R848 at 250 ng/ml synergistically increased phospho-I-κBα, phospho-p65, and nuclear translocation of phospho-p65, as well as decreased I-κBα indicating that this combination of stimulants synergistically activated the NF-κB signaling pathway in human MoDCs (Figures 4A,B). Furthermore, combined treatment activated phospho-p38, phospho-JNK, phospho-CREB, and phospho-c-Jun to a much greater extent than either treatment alone indicating that MAPK pathways were also synergistically activated in human MoDCs (Figure 4C). NF-κB and MAPK-signaling pathways promote the expression of many cytokines including that of IL-12p70 (4, 14, 30). These results suggest that synergistic stimulation by N1 and R848 of NF-κB and MAPK transcription factors contributed to the induction of IL-12p70, IL-1β, and TNF-α pro-inflammatory cytokines and maturation signals in human MoDCs.

### N1 Plus R848 Induce Type 1 IFN in Human MoDCs by Synergistic Activation of IRF3 and IRF7

**Figure 5:**
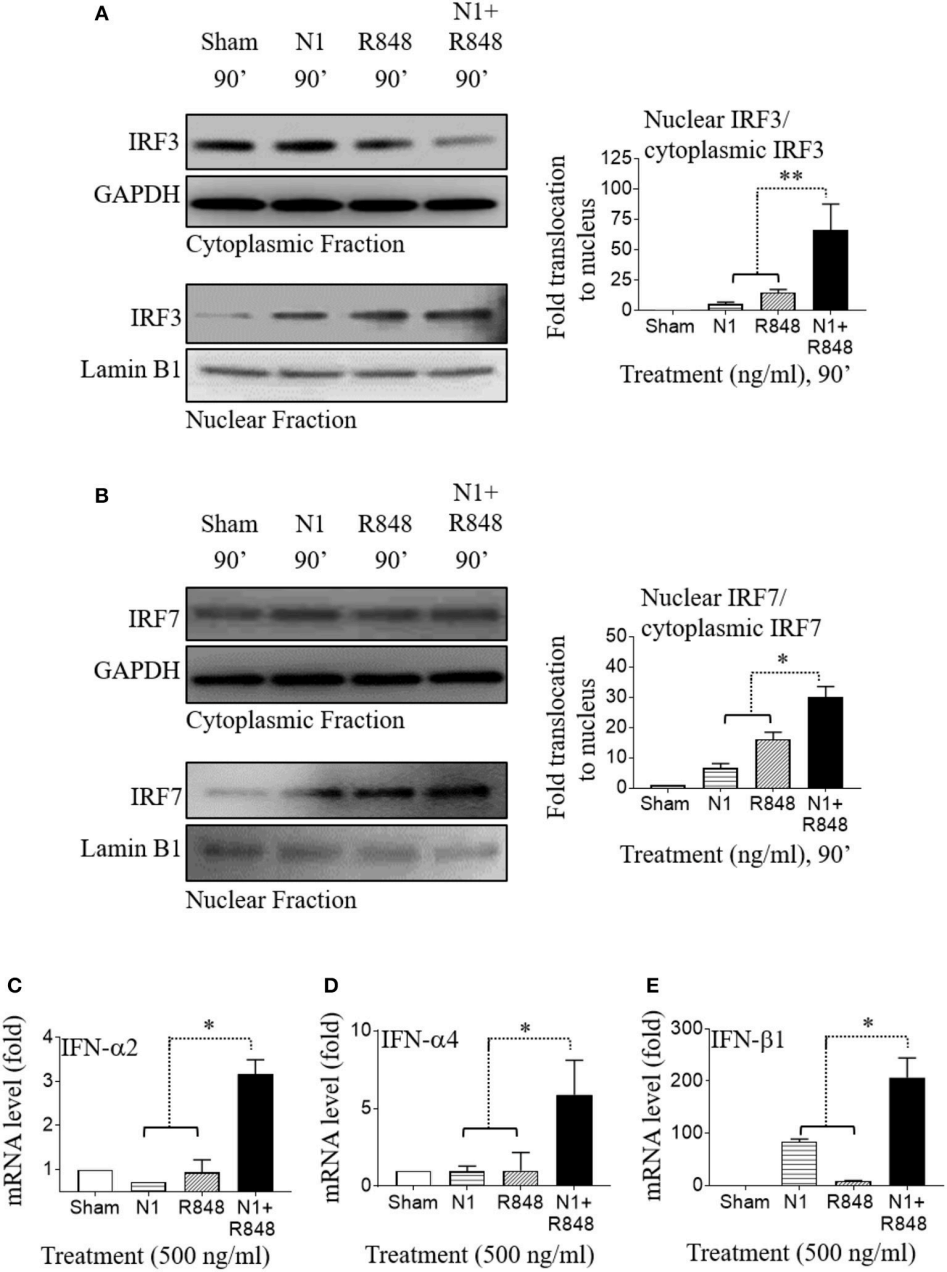
N1 and R848 synergistically activate IRF signaling as well as type 1 IFN responses in human MoDCs. **(A,B)** Serum-starved human MoDCs (2 × 10^6^ cells/ml) were treated for 0–90 min with N1 (500 ng/ml) and R848 (500 ng/ml) before they were solubilized in buffer (2 × 10^6^ MoDCs/0.1 ml) for analysis of the translocation of proteins from cytoplasmic to nuclear cell fractions. An identical amount of protein was loaded and separated by electrophoresis to detect the levels of **(A)** cytoplasmic fraction of IRF3, GAPDH, and nuclear fraction of IRF3, lamin B1 or **(B)** cytoplasmic fraction of IRF7, GAPDH, and nuclear fraction of IRF7, lamin B1. Band intensities from three donors was quantified and normalized with GAPDH or lamin B1 using ImageJ software. **(C–E)** Human MoDCs were treated with N1 (500 ng/ml) and/or R848 (500 ng/ml) for 6 h before extraction of total RNA. The levels of IFN-α2, IFN-α4, and IFN-β1 mRNA were quantitated by qPCR and shown as a fold increase over the sham treated DCs. Data are shown as the average (mean ± SD) of triplicates of one experiment representative of three. *p<*0.05*, and **p<*0.01* according to one-way ANOVA followed by Tukey's *post hoc* test.

In addition to the induction of pro-inflammatory genes, N1 and R848 stimulation of human MoDCs also showed a critical role in enhancing the production of type 1 IFN. To address whether N1 and R848 used common signaling pathways for inducing IFN in human MoDCs, we analyzed the activation status of IRF3 and IRF7 that have been described as a major transcription factors involved in IFN-α and IFN-β induction after their phosphorylation and translocation within the nucleus (35, 36). Human MoDCs treated with N1 (500 ng/ml) plus R848 (500 ng/ml) synergistically activated IRF3 as well as IRF7 transcription factors and increased their translocation to the nucleus (Figures 5A,B). This caused the robust activation of IFN-α2, IFN-α4, and IFN-β1 (Figures 5C–E) type 1 IFN in human MoDCs stimulated with N1 plus R848, which was greater than that induced by either single stimulant alone. Based on previous reports (8, 37), these data suggest that synergy between N1 plus R848 induced type 1 IFN through IRF3- and IRF7- dependent pathways that in turn enhanced the IL-12p70 production in activated human MoDCs. Furthermore, the CD4+ T cell expansion was also dependent on the signaling of type 1 IFN.

### N1 Dose Dependently Synergized With R848 in Stimulating Surface Marker Expression and Inducing Th1 Polarizing Cytokines Secretion by Mouse BMDCs

**Figure 6:**
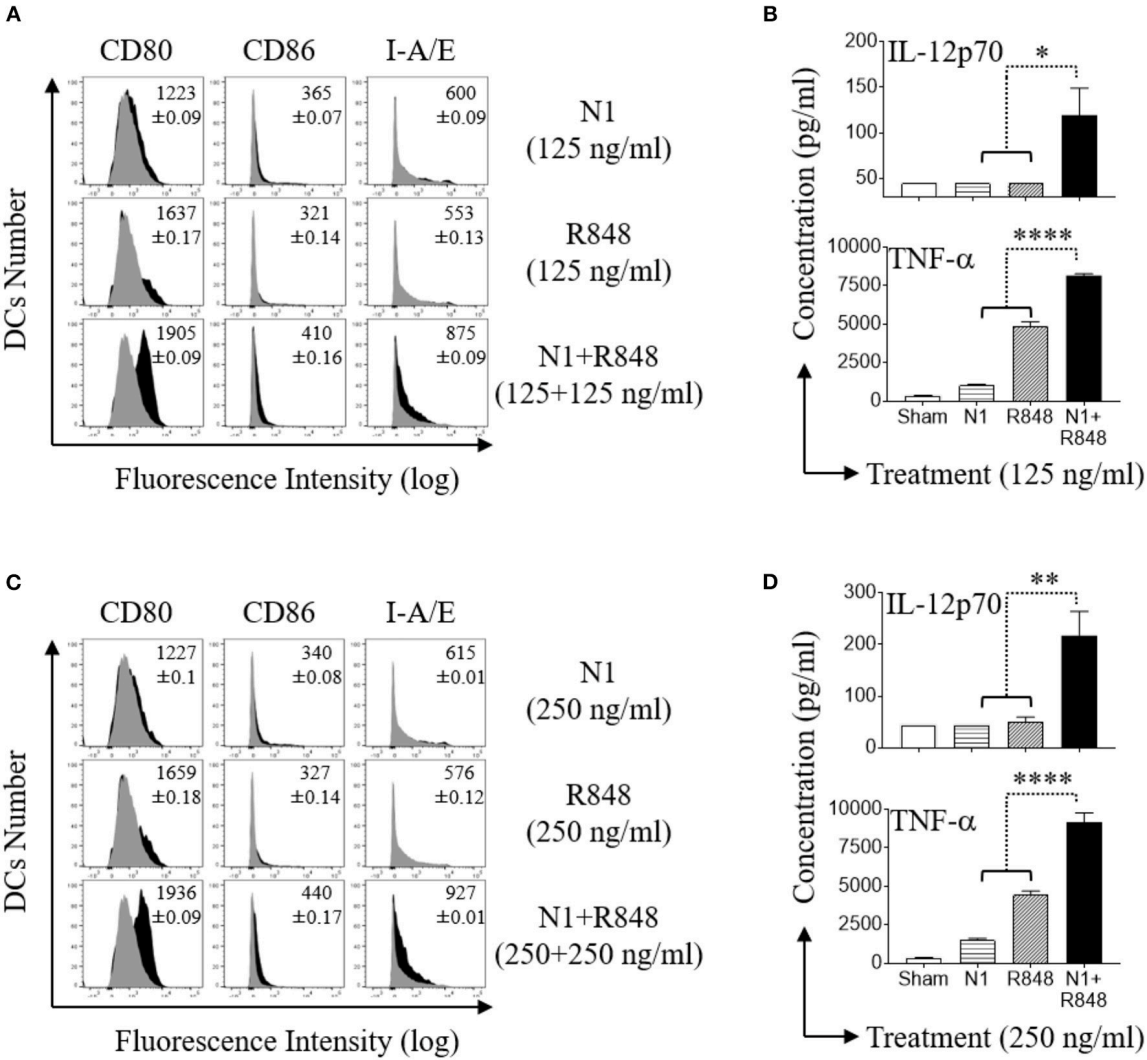
N1 and R848 synergistically induce phenotypic maturation of mouse BMDCs. **(A,C)** Mouse BMDCs were incubated at 5 × 10^5^ cells/ml in the absence (sham) or presence of N1 (125, 250 ng/ml) and R848 (125, 250 ng/ml) for 48 h before they were immuno-stained and analyzed by flow cytometry for the expression of the indicated surface molecules [inscribed numbers are the average (geometric mean fluorescence intensity ± SD) of three batches of male mice; gray = sham-treated]. **(B,D)** Cytokine levels in the culture supernatants for 48 h were quantitated by cytokine array (mean ± SD; *n* = 3). Shown are the results of one experiment representative of three from three different batches of male mice (*n* = 6). *p<*0.05*, **p<*0.01*, and ****p<*0.0001* according to one-way ANOVA followed by Tukey's *post hoc* test.

Mouse BMDCs express a functional TLR7 receptor which is triggered by R848 (38). Stimulation of mouse BMDCs with N1 and R848 in combination demonstrated considerable synergy (11). When treated with N1 (125 or 250 ng/ml) or R848 (125 or 250 ng/ml) by themselves, these ligands induced only very low levels of co-stimulatory (CD80 and CD86) and MHC class II (I-A/E) molecules on their surface (Figures 6A,C), as well as limited production of pro-inflammatory cytokine proteins (IL-12p70 and TNF-α) (Figures 6B,D). However, the combination of N1 with R848 dose dependently upregulated co-stimulatory molecules as well as pro-inflammatory cytokine production at levels much higher than either ligand alone. Thus, we confirmed that N1 dose dependently synergized with R848 in the activation of mouse BMDCs.

### NI Plus R848 Also Synergistically Activated NF-κB and IRF Signaling in Mouse BMDCs

**Figure 7:**
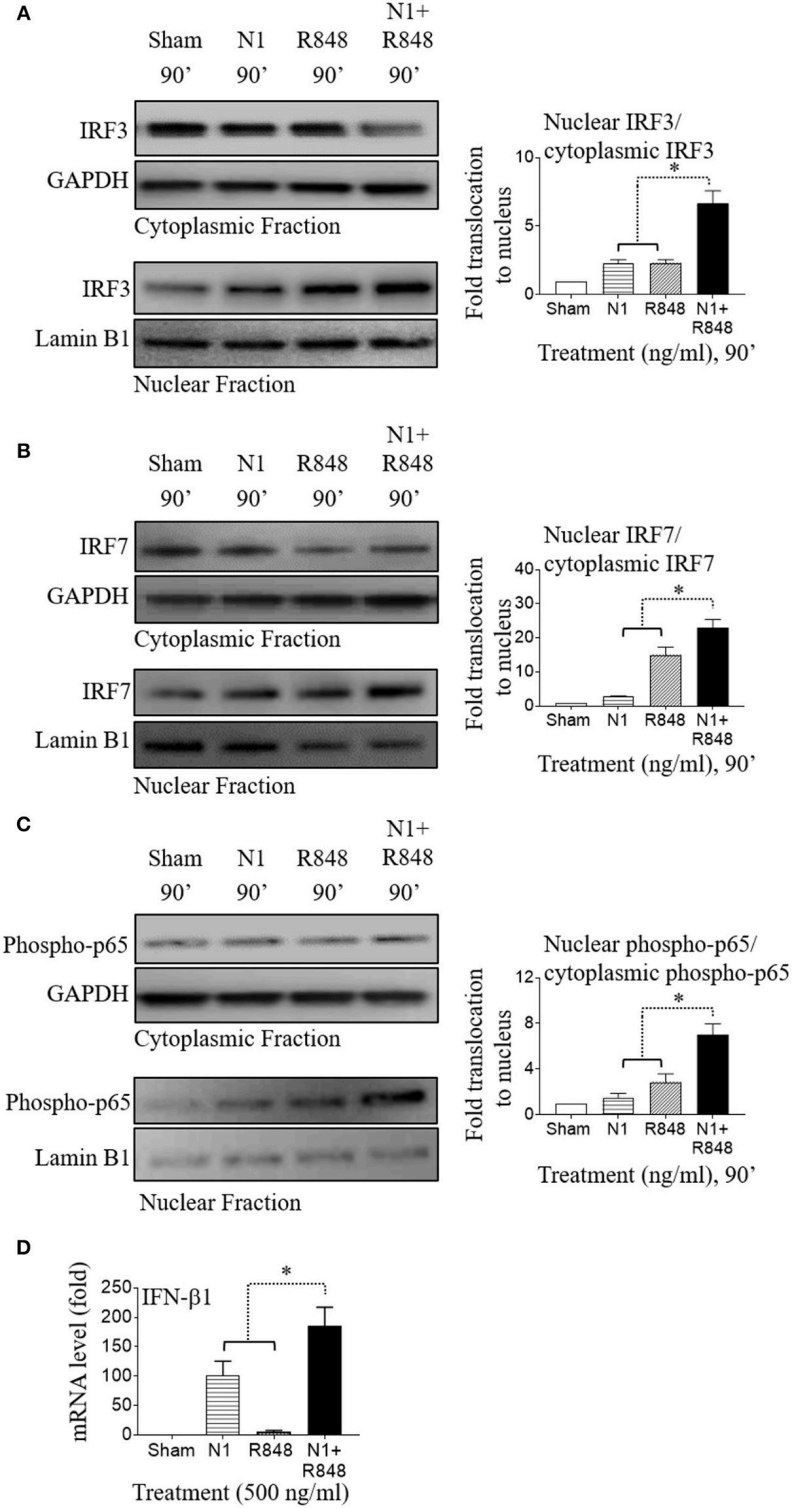
N1 and R848 synergistically activate NF-κB, IRF3, and IRF7 signaling as well as type 1 IFN responses in mouse BMDCs. **(A–C)** Mouse BMDCs (2 × 10^6^ cells/ml) were treated for 0–90 min with N1 (500 ng/ml) and R848 (500 ng/ml) before they were solubilized in the buffer for analysis of the translocation of proteins from cytoplasmic to nuclear cell fractions. An identical amount of proteins was loaded and separated by electrophoresis to detect the levels of cytoplasmic fraction of IRF3, IRF7, phospho-p65, and GAPDH, as well as nuclear fraction of IRF3, IRF7, phospho-p65, and lamin B1. Quantitation of band intensities from three different batches of male mice (*n* = 6) were normalized against GAPDH or lamin B1 using ImageJ software. **(D)** Mouse BMDCs were treated with N1 (500 ng/ml) and/or R848 (500 ng/ml) for 6 h before being used for the extraction of total RNA. The levels of IFN-β1 mRNA was quantitated by qPCR and shown as fold increase over the sham treated DCs. Data are shown as the average (mean ± SD) of triplicates of one experiment representative of three. *p<*0.05* according to one-way ANOVA followed by Tukey's *post hoc* test.

A previous study reported that NF-κB transcription factor regulates mouse BMDC activation and that inhibition of NF-κB activation blocked maturation of mouse BMDCs in terms of upregulation of MHC complex and co-stimulatory molecules (39). The transcription factors IRF3 and IRF7 coordinately regulate type 1 IFN induction in mouse BMDCs (40). IRF3 and IRF7 were also stimulated by TLR4 and TLR7/8 agonists and played an important role in the polarization of mouse BMDCs (41, 42). Therefore, we sought to determine whether pro-inflammatory cytokines and type 1 IFN production in mouse BMDCs by N1 plus R848 treatment also contributed to the proliferation of CD4+ T cells as well as their polarization into Th1 type cells. We treated mouse BMDCs with N1 (500 ng/ml) and R848 (500 ng/ml), and assessed the nuclear localization of phopho-p65, IRF3, and IRF7 transcription factors by Western blot. We found that these transcription factors were synergistically increased and translocated into the nucleus in mouse BMDCs when stimulated with N1 plus R848 (Figures 7A–C). As a result, type 1 IFN (IFN-β1) was also synergistically induced in mouse BMDCs stimulated with N1 plus R848 to a much greater extent than by each stimulant alone (Figure 7D). These results agree with another report by O'Neill (36) suggesting that NF-κB, IRF3, and IRF7 signaling pathways played an important role in the synergistic upregulation of pro-inflammatory cytokines as well as type 1 IFN production by mouse BMDCs.

### Synergistic Effect of N1 Plus R848 Treatment Also Revealed by RNA-seq Analysis

**Figure 8:**
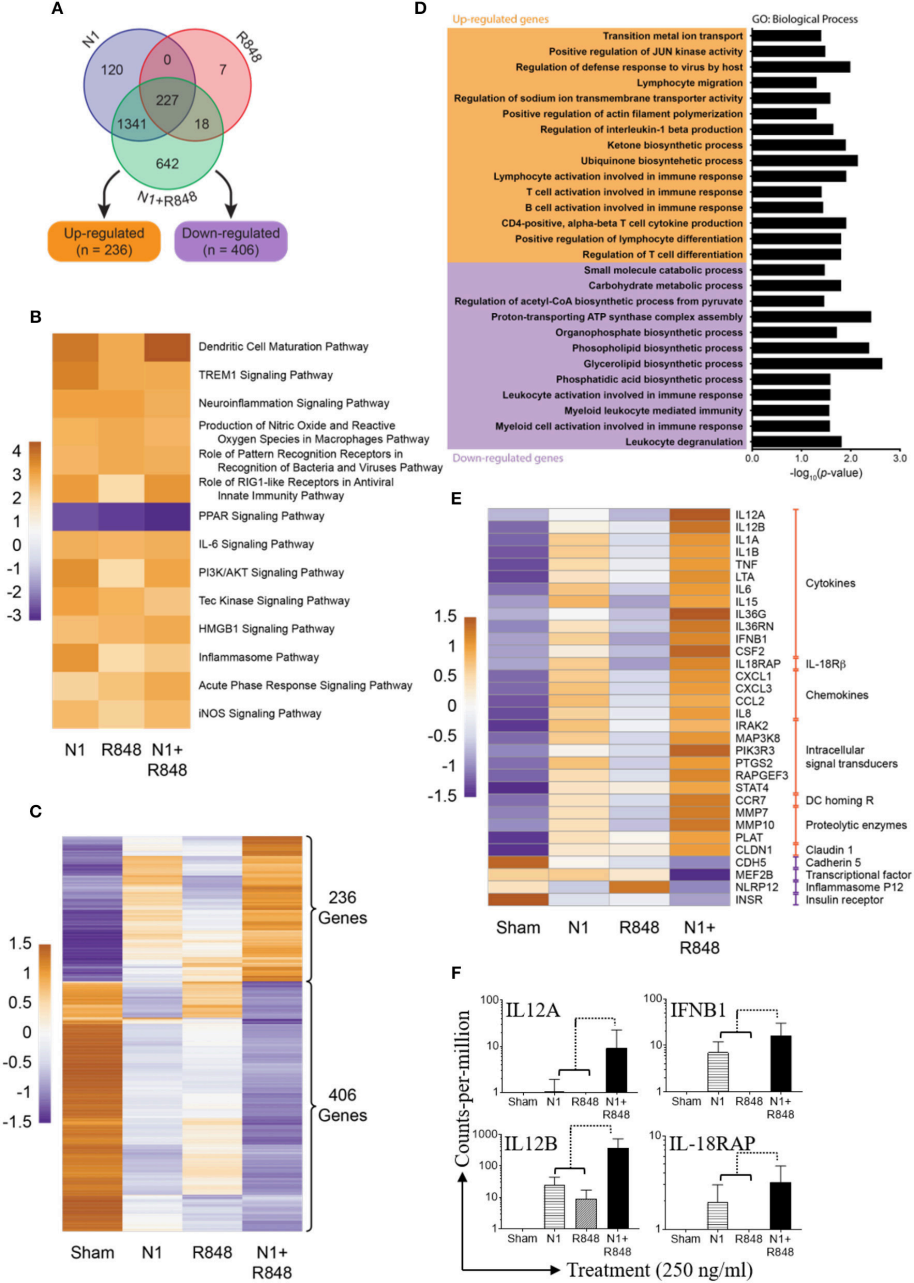
Synergy of N1 and R848 as revealed by RNA-seq analysis. Human MoDCs from three donors were stimulated with N1 (250 ng/ml) and/or R848 (250 ng/ml) for 4 h before extraction of total RNA. **(A)** Venn diagram of 2355 genes out of 14,284 genes were found to be variable (adjusted *p*<*0.05* and absolute fold change relative to sham ≥2.0). **(B)** Heat map showing pathways identified as significantly (absolute activation z-score ≥2.0) de/activated in all three experimental contrasts vs. sham. Cell values are activation z-scores and row centering has been applied. **(C)** Heat map showing the expression of 642 genes only found to be variable relative to sham when treated with N1 plus R848. **(D)** Results of gene ontology (GO) term analysis of the 642 genes exclusively variable in the N1 plus R848 treatment. Significant (*p*<*0.05*) GO terms for the up- and down-regulated subsets of these genes are given; the bar graph visualizes log_10_-transformed, Bonferonni-corrected *p*-values for each term. **(E)** Heat map showing the expression of 32 selected genes with relevance to DCs maturation. All genes in this subset were included in the set of 2,355 unique variable genes as well as the significantly de/activated pathway analysis**. (C,E)** Expression values in these heat maps are log_2_-transformed, TMM-normalized counts-per-million (CPM). **(F)** Shown are the fold expression of CPM of 4 genes selected from 28 synergistic upregulated genes obtained from three donors. Orange shows relatively high expression while purple shows relatively low expression. Row centering and unit variance scaling have been applied. Rows are clustered using Euclidean distances and complete linkage.

To examine the effect of N1 and R848 synergy on global gene expression, three human donor MoDCs were stimulated with sham, N1, R848, or N1 plus R848 for 4 h. Many genes were upregulated or downregulated by the stimulation and our analysis of RNA-seq datasets measured 14,284 genes in each of 12 samples (Figure S5A). After normalization and filtering, a total of 2,355 of these genes were found to be variable in at least one of the contrasts between an experimental treatment and sham (Figure S5B). Of these, 642 variable genes were perturbed only when N1 and R848 were both present, with 236 upregulated and 406 down-regulated relative to sham controls (Figures 8A,C). A gene ontology (GO) term analysis of these 642 genes revealed the majority to be involved in the synergistic upregulation of T-lymphocyte activation and differentiation pathways (Figure 8D). Pathway analysis revealed the DC maturation pathway as the most significantly activated in comparison with other activated pathways. In contrast, the peroxisome proliferator-activated receptor (PPAR) signaling pathway was most downregulated (Figure 8B). From the genes in these pathways, the most synergistically up- or downregulated 32 genes are in fact highly relevant to DC maturation, highlighting the synergistic effect of N1 plus R848 on DC maturation (Figure 8E). Of these 32 genes involved in DCs maturation, 28 gene transcripts that were synergistically upregulated involved cytokines, IL-18Rβ, chemokines, intracellular signal transducers, DC homing R, proteolytic enzymes, as well as claudin 1 signaling pathway. Four gene transcripts were highly synergistically downregulated, including cadherin 5, the MEF2B transcriptional factor, inflammasome P12 as well as insulin receptor signaling pathways (Figure 8E).

Out of 28 most upregulated transcripts in response to treatment with N1 plus R848, four genes including *IL12A, IL12B, IFNB1*, and *IL-18RAP* play critical roles in the polarization of CD4+ T lymphocytes into Th1 type cells. *IL12A*, a gene code for IL-12p35, was 1.2-fold upregulated by N1, not upregulated by R848, and 8.89-fold upregulated by N1 plus R848, indicating a clear synergistic effect between N1 and R848 (Figure 8F). *IL12B*, a gene coding for IL-12p40, was 27-fold upregulated by N1, 9-fold upregulated by R848, and 328-fold upregulated by N1 plus R848 (Figure 8F). This again demonstrated a clear synergistic effect between N1 and R848. This synergy was also shown by the expression levels of *IFNB1* and *IL-18RAP* in DCs stimulated with N1 and R848 alone or in combination (Figure 8F). Together with the data showing that treatment with N1 plus R848 synergistically induced IL-12p70 production in DCs (Figures 2, 6), it was clear that stimulation with N1 plus R848 triggered Th1-polarizing program in DCs. Therefore, RNA-seq data demonstrate that N1 plus R848 at the global gene expression level synergistically promotes DC maturation and subsequent upregulation of many genes in DCs that are involved in the polarization of CD4+ T cells into Th1-type effectors.

## Discussion

Conversion of tumor-infiltrating DCs from tolerogenic to immunogenic by providing the appropriate stimulation of maturation is critical for the induction of therapeutic antitumor immune responses characterized by the generation of IFN-γ-producing Th1 and CTL effectors (43). We have previously reported that administration of N1 and R848 into mouse tumors induces tumor-specific curative immunity (11, 44) as a result of stimulating robust maturation of tumor-infiltrating DCs (11). Indeed, stimulation by N1 and R848 resulted in the synergistic upregulation of DC surface markers, production of pro-inflammatory cytokines and antigen-presenting capacity (Figures 2, 3). Global gene expression analysis by RNA-seq further demonstrated that N1 and R848 acted in synergy to promote full maturation of human DCs. Most of the genes whose expression were highly synergistically upregulated by N1 plus R848 belong to the DC maturation pathway (Figure 8B). The expression of many genes was changed only in response to both N1 plus R848 (Figures 8A,C). Furthermore, the 28 genes most highly synergistically upregulated by N1 plus R848 express products characteristic of full DC maturation, including pro-inflammatory cytokines, chemokines, intracellular signal transducers, and the chemokine receptor CCR7 responsible for DC homing to draining lymph nodes, a hallmark of full DC maturation (Figure 8E). Thus, the combination of N1 and R848 synergize to promote the full maturation of human DCs, which reinforces the idea of using N1 and R848 which is able to induce curative antitumor immunity in mice (11, 44) as having the potential to be translated into human usage.

How do N1 and R848 synergize in inducing DC maturation and generation of Th1-polarizing signals? Stimulation of both human and mouse DCs with N1 plus R848 resulted in synergistic activation of NF-κB, MAPK, IRF3, and IRF7 signaling pathways (Figures 4A–C, 5A,B, 7A–C). These signaling pathways lead to the activation of many transcriptional factors (e.g., NF-κB, CREB, AP-1, IRF3, and IRF7), which, upon translocating to the nucleus, promote the transcription of genes characteristic of DC maturation/activation, including genes for cytokines (e.g., IL-12, IL-1β, and TNF-α), co-stimulatory molecules (CD80, CD83, and CD86), and MHC molecules (MHC class I and II). Furthermore, various transcription factors cooperate to synergistically upregulate the expression of genes characteristic of DCs activation, for example IL-12 upregulation requires both NF-κB and IRF (30, 45–47). Since N1 activates DCs predominantly by stimulating surface TLR4 and using both MyD88 and TRIF pathways (4), while R848 activates endosomal TLR7/8 using MyD88, but not the TRIF, pathway (14), it is likely that TLR4 stimulants can thus enhance the effects of TLR7/8 stimulants via the TRIF pathway. Therefore, cooperation of those pathways induced by N1 and R848 converging on the activation of various transcriptional factors presumably provides a key mechanism for their synergistic activation of DCs.

The second possible cause of the observed synergy may arise from the induction of type 1 IFN production and the subsequent positive feedback on DC activation. It was previously reported that activation of the IRF transcription factors downstream of TLR4 and TLR7/8 signaling result in the production of type 1 IFN (36). Synergy between TLR agonists is enabled by TLR4 mediated IRF3-initiated production of IFNβ1, which in turn also leads to higher IRF7 transcription factor synthesis. This positive feedback can result in a more efficient TLR7/8 signaling (8, 48). In our study, N1 plus R848 stimulation of DCs promoted the synergistic production of both IRF3 and IRF7 leading to greater secretion of type 1 IFN such as IFN-α2, IFN-α4, and IFN-β1 than by single agonists alone (Figures 5C–E, 7D, 8E,F). Therefore, as shown by us and others induction of type 1 IFN plays an important role in furthering the cascade of gene expression after TLR signaling (49) and an autocrine loop of type 1 IFN contributes to enhanced production of bioactive pro-inflammatory cytokines such as IL-12p70 (8).

A third possible cause of the synergy of DC activation and immune responses induced by N1 and R848 can arise from the activation of distinct subsets of DCs by N1 and R848. In both humans and mice, TLR4 and TLR7/8 are expressed by different subsets of DCs. pDCs selectively express TLR7/8 but not TLR4, whereas cDCs express functional TLR4, but not TLR7/8 (43, 50–53). R848, by engaging TLR7/8 in the endosomes, selectively activates pDCs, leading to the production of type 1 IFN. N1, by triggering TLR4 on cell surfaces, activates cDCs (but not pDCs), promoting antigen uptake, processing, presentation, and the production of pro-inflammatory cytokines (4, 43, 52, 54). Therefore, R848 induced activation of pDCs through TLR7/8 in turn activates immature tumor-infiltration cDCs to become able to induce antitumor immunity. Thus, activation of distinct subsets of DCs by N1 and R848 can potentially result in more robust synergistic activation of DCs, particularly *in vivo*.

It has previously been reported that there is synergy between TLR4 agonist LPS and TLR7/8 agonist R848 in inducing a Th1-polarizing program in DCs (30). However, N1 is different from other TLR4 agonists such as LPS since it interacts with MD2 of the TLR4 receptor complex at a site that is different from where LPS binds (4), suggesting that there might be difference between N1+R848-induced and LPS+R848-induced DC maturation. In addition, N1 as an alarmin is endogenously produced and likely to have less deleterious effect than LPS in *in vivo* usage, which is particularly relevant in the context of implication for anticancer immunotherapy. We have in fact recently demonstrated that N1 and R848 administered in combination into established mouse tumors promoted more robust *in vivo* maturation/activation of tumor-infiltrating DCs (11) and consequently promoted greater antitumor immunity than either one alone in multiple mouse tumor models (11, 44). The possibility of utilizing the combination of N1 and R848 to promote therapeutic antitumor immunity in humans is being evaluated. N1 and R848 do synergize to induce the maturation/activation of human DCs in a way that potentially promotes the generation of beneficial human antitumor immune responses. Indeed, human DCs treated with N1 plus R848 preferentially stimulated the production of IFN-γ and upregulation of T-bet by CD4+ T cells in an allogeneic MLR (Figure 3).

In conclusion, the current study has demonstrated that stimulation by N1 plus R848 results in remarkable synergistic activation of DCs through NF-κB, MAPK, IRF3, and IRF7 signaling pathways. These signaling pathways promoted the synergistic production of Th1 polarizing cytokines, most importantly IL-12p70, TNF-α, and IL-1β, IL-18RAP, as well as type 1 IFNs (IFN-α and IFN-β1). This in turn led to the enhanced expression of transcription factor T-bet and synergistic IFN-γ production for synergistic expansion of Th1 type polarization. These findings strongly support the hypothesis that the combined stimulation of DCs by N1 plus R848 result in enhanced induction of Th1 immune responses, which is highly desirable for immunotherapeutic as well as antitumor vaccination strategies.

## Ethics Statement

All experiments with mice were performed in compliance with the principles and procedures outlined in the National Institutes of Health Guide for the Humane Care and Use of Animals and were approved by the NCI at Frederick Animal Care and Use Committee.

## Materials Availability

Recombinant human HMGN1 can be obtained through an MTA.

## Author Contributions

MA, DY, and JO for Conception and design. MA, DY, and AT for Development of methodologies and Acquisition of data. MA, DY, TM, and JO for Analysis and interpretation of data, as well as Writing, review, and/or revision of the manuscript. AT and DY for Administrative or technical support. DY and JO for Study supervision.

### Conflict of Interest Statement

The authors declare that the research was conducted in the absence of any commercial or financial relationships that could be construed as a potential conflict of interest.
