# Supplementary material for: HMGN1 and R848 Synergistically Activate Dendritic Cells Using Multiple Signaling Pathways
Source: Front Immunol. 2018 Dec 18;9:2982. doi: 10.3389/fimmu.2018.02982 (PMC6305469; doi:10.3389/fimmu.2018.02982)
Supplement: Supplementary file 1 [file Data_Sheet_1.pdf]

Supplementary Material

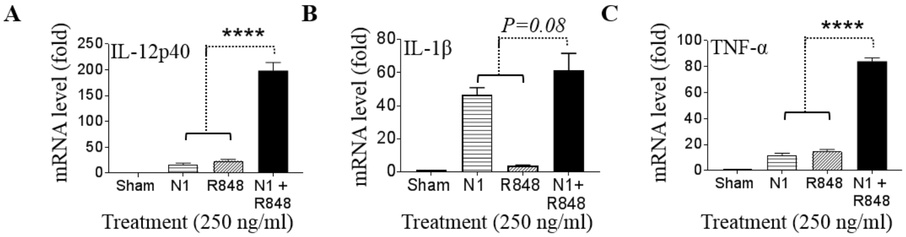

Figure S1. Human MoDCs at 5 x 10^5 cells/ml were treated with N1 (250 ng/ml) and/or R848 (250 ng/ml) for 6 h before extraction of total RNA. The levels of **(A)** IL-12p40, **(B)** IL-1β, and **(C)** TNF-α were quantitated by qPCR and shown as a fold increase over the sham treated basal level. Data are shown as the average (mean ± SD) of triplicates of one experiment representative of three. ****$p < 0.0001$ according to one-way ANOVA followed by Tukey's post hoc test.

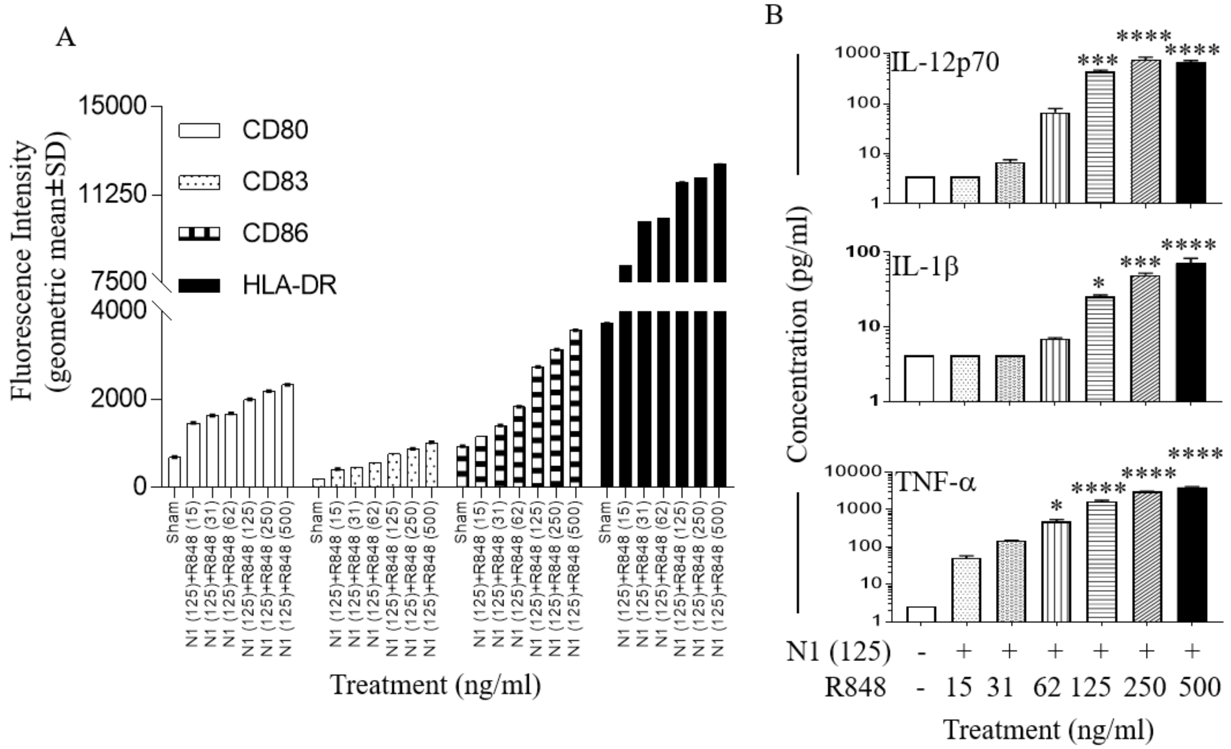

Figure S2. N1 and R848 synergistically induce phenotypic maturation of human MoDCs. Human MoDCs were incubated at 5 x 10^5 cells/ml in the absence (sham) or presence of N1 (125 ng/ml) and R848 (15 to 500 ng/ml) for 48 h before **(A)** they were immuno-stained and analyzed by flow cytometry for the expression of the indicated surface molecules (geometric mean fluorescence intensity ± SD from three donors). **(B)** cytokine levels in triplicate culture supernatants of one representative experiment of three performed from three donors with similar results were quantitated by cytokine array (mean ± SD). $p < 0.05$ is considered statistically significant when

compared with sham treatment. *$p < 0.05$, ***$p < 0.001$, and ****$p < 0.0001$ according to one-way ANOVA followed by Tukey's post hoc test.

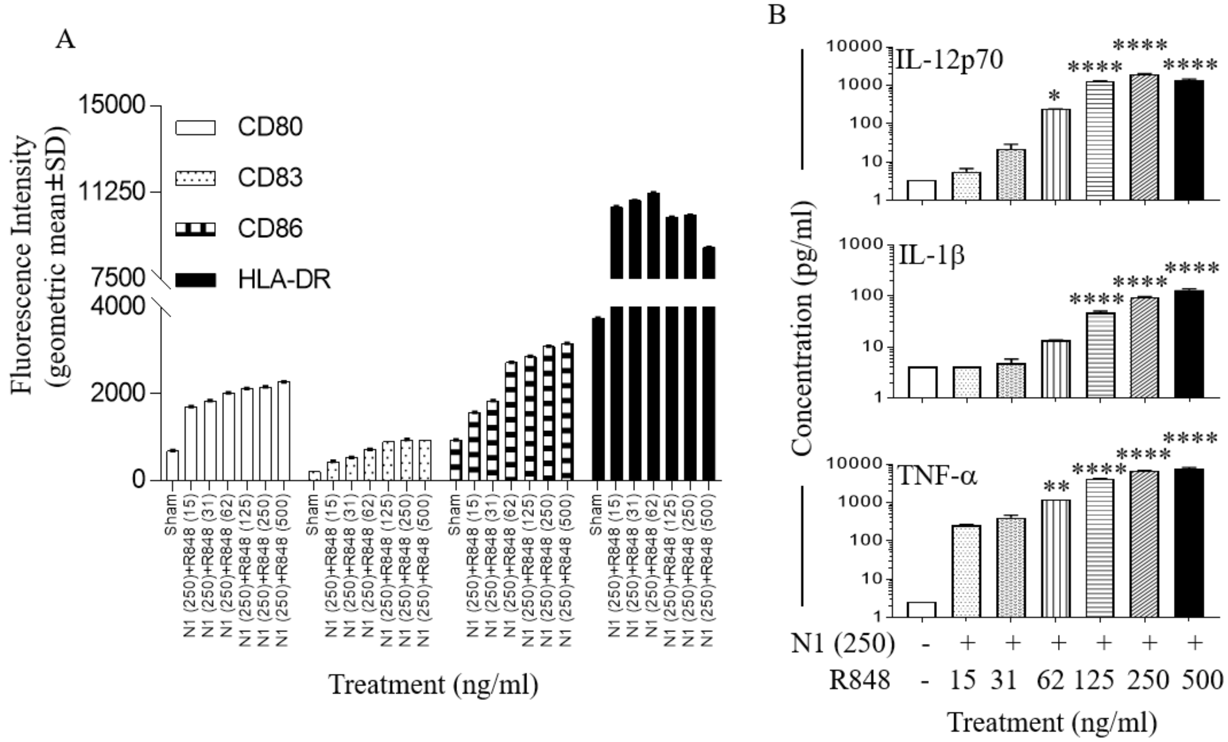

Figure S3. N1 and R848 synergistically induce phenotypic maturation of human MoDCs. Human MoDCs were incubated at 5 x 10^5 cells/ml in the absence (sham) or presence of N1 (250 ng/ml) and R848 (15 to 500 ng/ml) for 48 h before **(A)** they were immuno-stained and analyzed by flow cytometry for the expression of the indicated surface molecules (geometric mean fluorescence intensity ± SD from three donors). **(B)** cytokine levels in triplicate culture supernatants of one representative experiment of three performed from three donors with similar results were quantitated by cytokine array (mean ± SD). $p < 0.05$ is considered statistically significant when compared with sham treatment. *$p < 0.05$, **$p < 0.01$, and ****$p < 0.0001$ according to one-way ANOVA followed by Tukey's post hoc test.

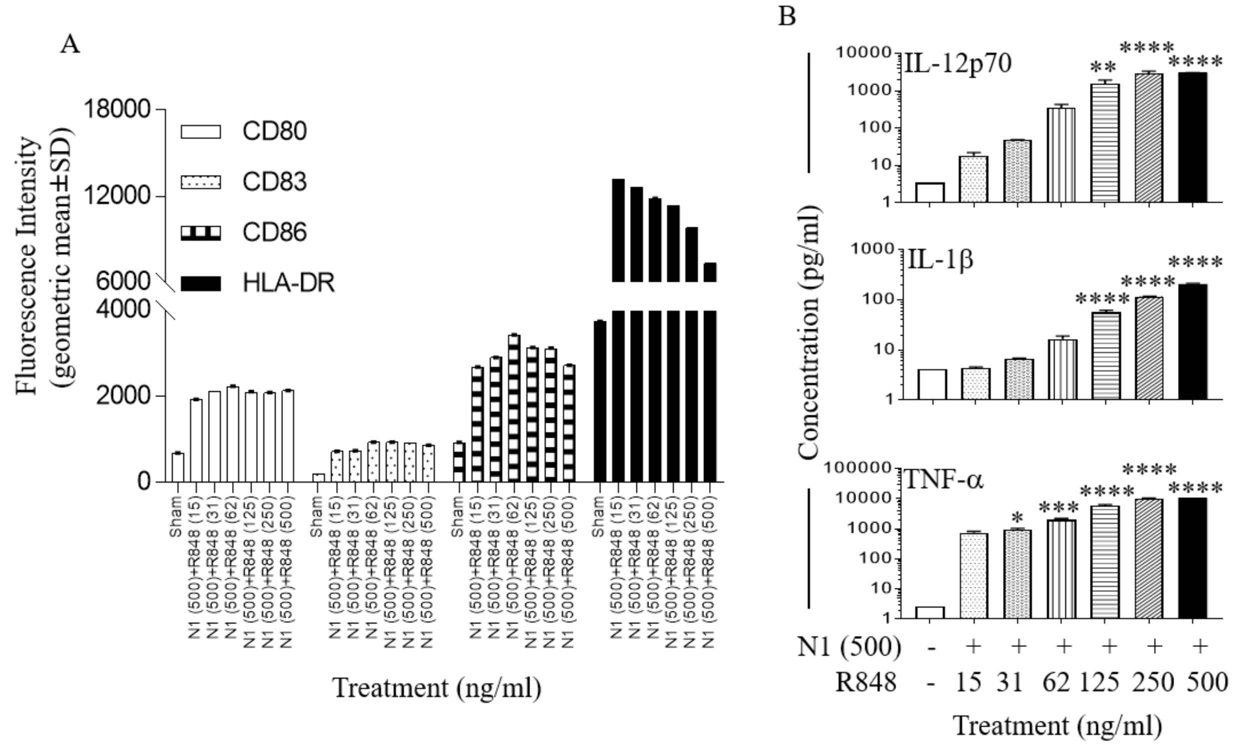

Figure S4. N1 and R848 synergistically induces phenotypic maturation of human MoDCs. Human MoDCs were incubated at 5 x 10^5 cells/ml in the absence (sham) or presence of N1 (500 ng/ml) and R848 (15 to 500 ng/ml) for 48 h before **(A)** they were immuno-stained and analyzed by flow cytometry for the expression of the indicated surface molecules (geometric mean fluorescence intensity ± SD from three donors). **(B)** cytokine levels in triplicate culture supernatants of one representative experiment of three performed from three donors with similar results were quantitated by cytokine array (mean ± SD). $p < 0.05$ is considered statistically significant when compared with sham treatment. *$p < 0.05$, **$p < 0.01$, ***$p < 0.001$ and ****$p < 0.0001$ according to one-way ANOVA followed by Tukey's post hoc test.

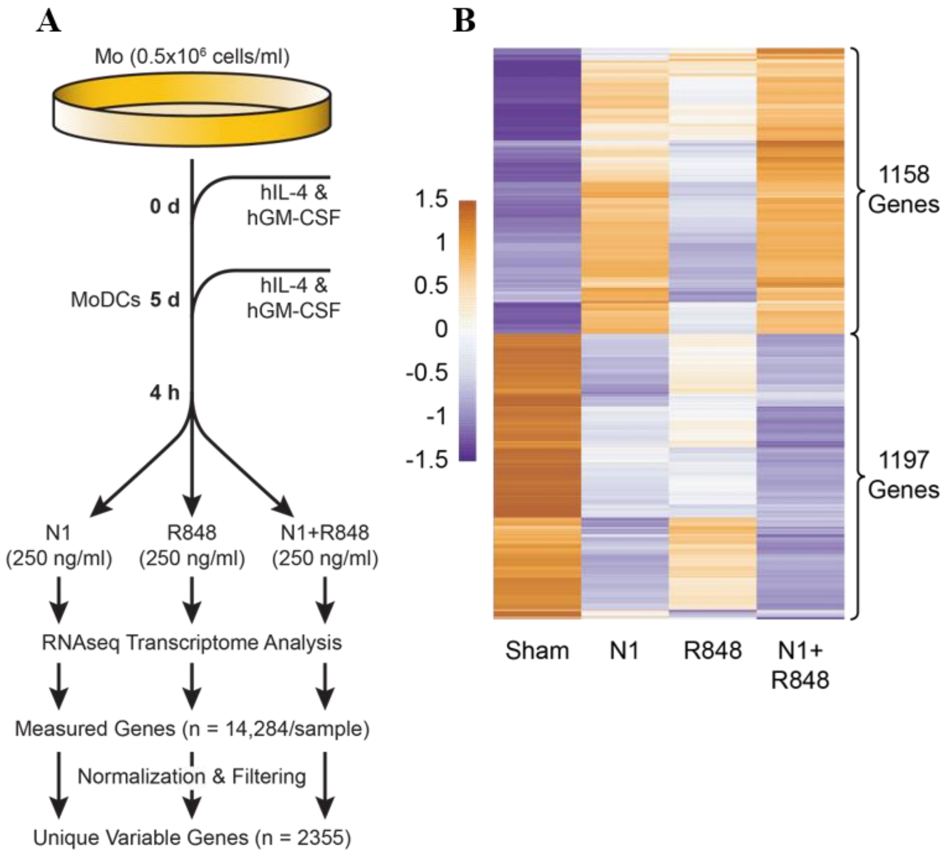

Figure S5. Response to N1 and R848 as revealed by RNA-seq analysis. **(A)** Workflow of RNA-seq analysis of MoDCs from three donors. MoDCs from each donor were prepared as described in manuscript and treated with N1, R848, and N1 plus R848, as well as sham. **(B)** Heat map showing the expression of 2,355 unique genes found to be variable in experimental treatments relative to sham. Expression values in these heat maps are $\log_2$-transformed, TMM-normalized counts-per-million (CPM). Orange shows relatively high expression while purple shows relatively low expression. Row centering and unit variance scaling have been applied. Rows are clustered using Euclidean distances and complete linkage.

Supplementary Table S1. qPCR primers for human and mouse cytokine genes.

Target genes	Catalog	Reference
Human IFN-α2	PPH00379A-200	NM_000605
Human IFN-α4	PPH01067B-200	NM_021068
Human IFN-β1	PPH00384F-200	NM_002176
Human IL-1β	PPH00171C-200	NM_000576
Human TNF-α	PPH00341F-200	NM_000594
Human IL-12p40	PPH00545A-200	NM_002187
Human β-actin	PPH00073G-200	NM_001101
Mouse IFN-β1	PPM03594C-200	NM_010510
Mouse β-actin	PPM02945B-200	NM_007393

The qPCR primers were obtained from Qiagen.
